# Risk factors for repeated dexamethasone intravitreal implant therapy for macular edema due to treatment-naïve branch retinal vein occlusion

**DOI:** 10.1186/s12886-021-01904-8

**Published:** 2021-03-20

**Authors:** Yu-Te Huang, Chun-Ju Lin, Huan-Sheng Chen, Peng-Tai Tien, Chun-Ting Lai, Ning-Yi Hsia, Jane-Ming Lin, Wen-Lu Chen, Yi-Yu Tsai

**Affiliations:** 1 Department of Ophthalmology, China Medical University Hospital, 2 Yuh-Der Road, Taichung City, Taiwan 40447; 2 School of Medicine, College of Medicine, China Medical University, Taichung, Taiwan; 3 Department of Optometry, Asia University, Taichung, Taiwan; 4 An-Shin Dialysis Center, NephroCare Ltd., Fresenius Medical Care, Taichung, Taiwan; 5 Graduate Institute of Clinical Medical Science, China Medical University, Taichung, Taiwan

**Keywords:** Age, Branch retinal vein occlusion, Central retinal thickness, Dexamethasone intravitreal implant, Diabetes, Macular edema

## Abstract

**Background:**

This study evaluated the effects of dexamethasone intravitreal implant on treatment-naïve branch retinal vein occlusion (BRVO)-induced macular edema (ME), and the risk factors for earlier repeated treatment.

**Methods:**

Patients treated from 2013 to 2016 were enrolled. The patients’ demographics, medical history, best-corrected visual acuity (BCVA), and central retinal thickness (CRT) were recorded. Risk factors for repeated treatment were identified using a Cox proportional hazard model and logistic regression.

**Results:**

29 patients (mean age: 58.64 ± 13.3 years) were included; 44.8% received only one injection, while 55.2% received two or more. The mean initial CRT was 457.8 ± 167.1 μm; the peak CRT and final CRT improved significantly to 248.9 ± 57.9 μm and 329.2 ± 115.1 μm, respectively. The peak BCVA improvement and final improvement were 29.5 ± 23.5 approximate ETDRS letters and 19.8 ± 24.4 letters, respectively, with 62.1% of patients improving by more than 15 letters. Older age, higher initial CRT, and diabetes were the risk factors for multiple injections.

**Conclusion:**

Dexamethasone intravitreal implant results in significant peak CRT and BCVA improvements, while older age, higher initial CRT, and diabetes are risk factors for repeated injections. The optimal retreatment schedule for these patients should be further explored.

## Background

Retinal vein occlusion (RVO) is second only to diabetic retinopathy in terms of prevalence among retinal vascular disorders and is a major cause of vision loss worldwide [1–4]. Older age is known to be a major risk factor for the disorder, with a meta-analysis by Rogers et al. [2] of RVO in various regions of the world showing a prevalence of only 1.57/1000 among 40- to 49-year-olds versus a prevalence of 12.76/1000 in 70- to 79-year-olds. Broadly speaking, the disorder typically occurs in people aged older than 50 years [5], and as this age group continues to grow due to ongoing demographic trends [6], the number of individuals afflicted with RVO is likewise expected to increase.

Branch RVO (BRVO) can result in numerous complications, including macular edema (ME), retinal neovascularization, retinal detachment, and vitreous haemorrhage [1]. ME is the most common complication, with approximately 5–15% of eyes with BRVO developing ME within one year, and also the single most important cause of vision loss [7]. Although roughly 18–41% of BRVO-induced ME (BRVO-ME) cases resolve spontaneously over time [2], the extended period of hypoxia resulting from ME can cause irreversible losses of visual acuity even in such cases, while those cases that do not resolve spontaneously can be even more damaging and may thus call for treatment [1]. In fact, because of the impacts of ME on quality of life (QoL) – one study found that RVO-ME-induced vision loss causes meaningful declines in several aspects of health-related QoL [8] – most BRVO-ME patients are willing to undergo invasive treatments in spite of the possibility of spontaneous resolution [9].

Most anti-vascular endothelial growth factor (VEGF) agents for BRVO require frequent dosing due to their relatively short half-lives. In contrast, the dexamethasone intravitreal implant (Ozurdex) reportedly has a longer duration of up to 4–6 months. The present study thus evaluated the effects of Ozurdex, including its safety and duration of action, in treatment-naïve BRVO-ME cases. In addition, we tried to identify possible risk factors for earlier repeated treatment after Ozurdex treatment according to the evidence of disease flare-ups in spectral-domain optical coherence tomography (OCT) images of the same BRVO-ME patients.

## Methods

This retrospective, interventional case series study included patients treated from January 1, 2013, to December 31, 2016, at China Medical University Hospital in Taiwan. We launched a project to prospectively add patients with BRVO-ME to the study group. After treating them under a strict treatment regimen and follow-up protocol, we then retrospectively reviewed and included the patients in our study. More specifically, we selected only those patients from that time period who met the following strict criteria: 1) a diagnosis of ME secondary to BRVO confirmed by three senior retinal subspecialists (CJ Lin, PT Tien, and CT Lai) and with a baseline central retinal thickness (CRT) of more than 300 μm; 2) vision loss resulting from ME after BRVO of less than 6 weeks’ duration; 3) OCT graders who were blinded to the treatment received by the patient; 4) an intravitreal implant of 0.7 mg dexamethasone (Ozurdex) given as the baseline treatment; 5) treatment with at least one injection of Ozurdex with follow-up visits lasting at least 6 months; and 6) all follow-up visits conducted on schedule unless the patient was lost to follow-up.

The main exclusion criteria, meanwhile, were as follows: 1) a history of pars plana vitrectomy in the study eye; 2) concomitant glaucoma; 3) a history of diabetic retinopathy including diabetic ME; 4) a pre-existing macular pathology, such as age-related macular degeneration, macular hole, or macular pucker; and 5) a history of use of steroids via other means, of laser treatment, or of intravitreal anti-VEGF injection for BRVO before receiving Ozurdex. Furthermore, 6) for those patients younger than 50 years old, an internist referral was arranged, and the given patient was excluded if certain underlying autoimmune diseases were diagnosed. Finally, 7) a patient was excluded if fluorescein angiography (FA) showed the presence of more than 10 disc areas of retinal non-perfusion (ischemic BRVO). The Institutional Review Board of China Medical University Hospital approved the study protocol, and the study was performed in accordance with the World Medical Association’s Declaration of Helsinki. Written informed consent was obtained from all of the patients who were ultimately included.

For each Ozurdex injection, the medication was injected intravitreally via the pars plana (3.5 mm away from the limbus). According to the 2018 Euretina Expert Consensus Recommendations guidelines, injections should be made between 3.5 and 4 mm from the limbus [10]. In addition, the AAO has suggested a distance of 3.5 to 4 mm posterior to the limbus for a phakic eye [11]. We used the lower limit according to the two consensuses for a reason. Specifically, the patients in this study were all of Asian ethnicity, and the lens position is typically closer to anterior segments in Asian individuals [12]. In fact, 3.5 mm has been the standard protocol distance in our hospital for many years, and no traumatic cataracts have occurred thus far as a result. After the injection, the intraocular pressure (IOP) and retinal artery perfusion were checked, and the patient received topical levofloxacin four times daily for 7 days.

All of the patients were followed up on a monthly schedule. Each patient’s demographic data, medical history (including diabetes and hypertension), ocular diagnosis, best-corrected visual acuity (BCVA, as determined by approximate Early Treatment Diabetic Retinopathy Study (ETDRS) letter scores) [13], IOP, and CRT as determined by OCT (Spectralis, Heidelberg, Germany), as well as the occurrence of any complications, were noted in and later retrieved from electronic medical records completed every month throughout the study period. Any patients whose IOP exceeded 25 mmHg at any visit was evaluated and treated accordingly. Patients were eligible for retreatment with Ozurdex if their retinal thickness increased by 50 μm from the lowest recorded level, and further doses of Ozurdex were also given if the patient experienced a recurrence of ME as determined by OCT.

The time to additional Ozurdex treatment was analysed using Kaplan-Meier analysis, and the possible risk factors for retreatment (single injection vs. multiple injections) were identified using multivariate Cox proportional hazard analysis. We also tried to come up with a reference cut-off value to define high-risk patients for multiple infections. Multivariate logistic regression analysis was used to verify the most significant risk factor and the value of the risk elevation.

## Results

A total of 29 patients were ultimately included in this study (Table 1). Their mean duration of follow-up was 23.3 ± 15.9 months, and the longest duration of follow-up time was 60 months. Sixteen patients (55.2%) were male, 13 (44.8%) were female, and they had a mean age of 58.64 ± 13.3 years.

**Table 1:** Baseline demographics of all study participants

	All BRVO Patients (***n*** = 29)
Age (range, years)	58.6 ± 13.3 (25 ~ 83)
Gender (%)	
Female	13 (44.8%)
Male	16 (55.2%)
Diabetes (%)	12 (41.4%)
Hypertension (%)	16 (55.2%)
Eye (%)	
OD	14 (48.3%)
OS	15 (51.7%)
BCVA, letters (range)	40.9 ± 31.4 (−15 ~ 85)
CRT (range, μm)	457.8 ± 167.1 (229 ~ 787)
IOP (range, mmHg)	15.3 ± 3.3 (8 ~ 21)
Lens Status (%)	
Phakic	21 (72.4%)
Pseudophakic	8 (27.6%)
Follow-up (range, months)	23.3 ± 15.9 (6 ~ 60)

*BCVA* best-corrected visual acuity, *CRT* central retina thickness, *IOP* intraocular pressure

**Fig. 1:**
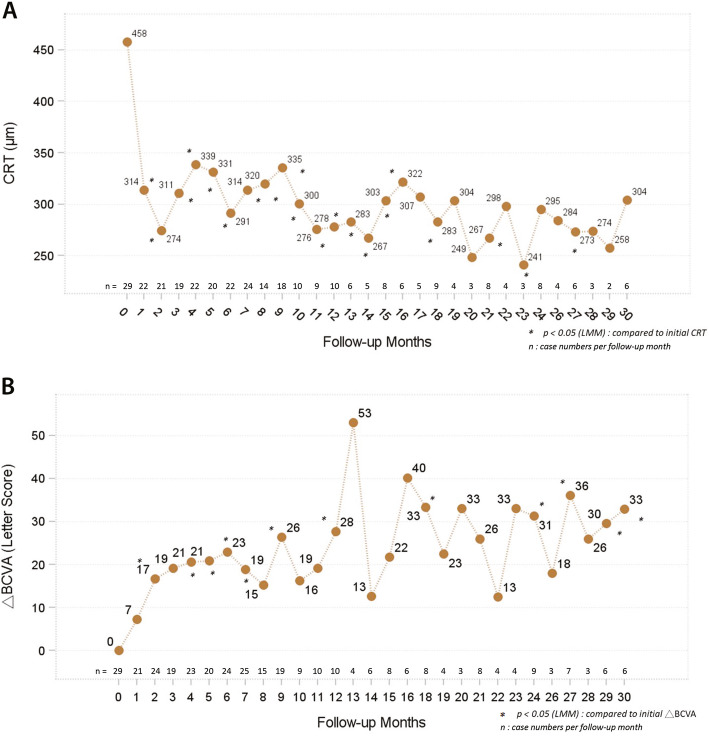
**a** CRT showed rapid improvement in the first month. **b** The improvement in BCVA was significant after 2 months

The mean peak change in CRT for all 29 patients after the Ozurdex treatments reached a statistically significant level (dropping from a mean initial CRT of 457.8 ± 167.1 μm to a lowest mean CRT of 248.9 ± 57.9 μm, *p* < 0.0001). The mean final change in CRT after the treatments was 128.6 μm (p < 0.0001). Overall, 24.1% of the patients achieved a final CRT of less than 250 μm after the treatments, and 58.6% achieved a final CRT of less than 300 μm. During the follow-up period, CRT showed rapid improvement in the first month, then fluctuated within a stable range (shown in Fig. 1a).

The mean peak change in BCVA (as determined by approximate ETDRS letter scores) of all 29 patients after the treatments was 29.5 ± 23.5 letters (*p* < 0.0001), and the mean final change was 19.8 ± 24.4 letters (*p* < 0.0002). The mean improvement in BCVA was significant after 2 months (shown in Fig. 1b), taking slightly longer to reach the level of significance than the CRT changes.

During the follow-up period, 13 (44.8%) of the patients only received one injection and 16 (55.2%) received two or more injections. Among those 16 patients, only one (3.5% of the total of 29 patients) received six doses due to recurrent ME, while the remaining 15 patients received two doses. A comparison of the single-injection group with the multiple-injection group revealed that the average age and proportion with diabetes mellitus were significantly higher in the multiple-injection group (Table 2).

**Table 2:** Baseline data comparison between single- and multiple-injection groups

	All Patients n = 29	Single-injection ***n*** = 13 (44.83%)	Multiple-injection ***n*** = 16 (55.17%)	***P***^***1***^
Age (in years)	58.59 ± 13.27	51.08 ± 13.41	64.69 ± 9.82	*0.003
Gender				
Female	13 (44.83%)	4 (30.77%)	9 (56.25%)	*0.264*
Male	16 (55.17%)	9 (69.23%)	7 (43.75%)	
DM	12 (41.38%)	2 (15.38%)	10 (62.50%)	*0.021
Hypertension	16 (55.17%)	7 (53.85%)	9 (56.25%)	*1.000*
BCVA (Letters)	40.93 ± 31.41	47.69 ± 31.78	35.44 ± 31.01	*0.300*
CRT (in μm)	457.8 ± 167.1	391.2 ± 135.0	511.9 ± 174.9	*0.051*
IOP (in mmHg)	15.31 ± 3.31	15.31 ± 2.95	15.31 ± 3.66	*0.990*
Lens Status				
Phakic	19 (65.52%)	11 (84.62%)	8 (50.00%)	*0.114*
Pseudophakic	10 (34.48%)	2 (15.38%)	8 (50.00%)	

^1^Comparing single-injection with multiple-injection groups; * *P* < 0.05

*BCVA* best-corrected visual acuity, *CRT* central retina thickness, *IOP* intraocular pressure

In order to determine what factors might influence the interval until the second treatment among those in the multiple-injection group, some of the initial conditions of the 16 patients who received more than one injection were analysed. Using the second treatment with Ozurdex as a final event, a Kaplan–Meier analysis revealed that the median time to the second treatment for all 16 patients was 7.03 months.

**Fig. 2:**
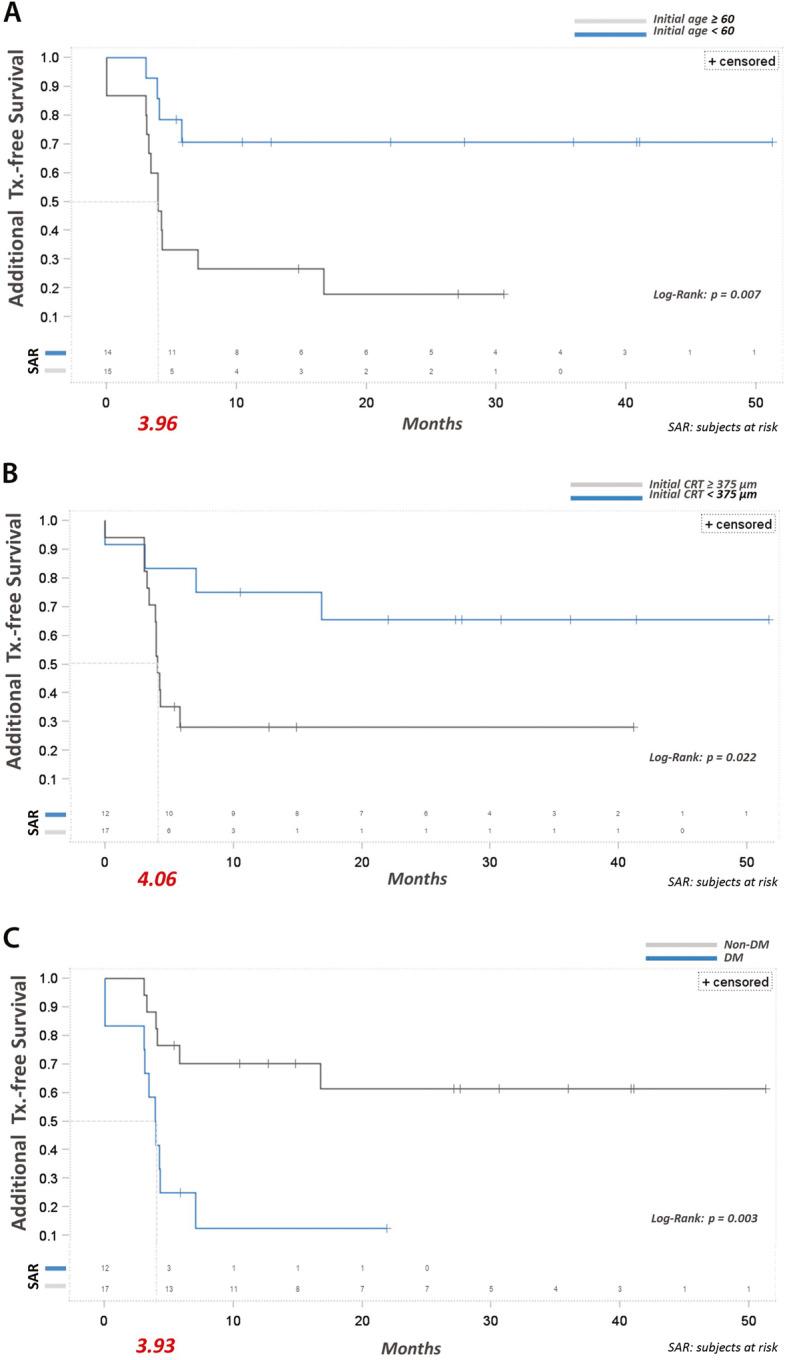
**a** Kaplan–Meier analysis stratified showed that the median time to the second Ozurdex treatment in the group older than 60 years was 3.96 months, whereas the median time to second treatment in the group younger than 60 years was greater than 50 months (p = 0.007). **b** The median time to the second Ozurdex treatment in the initial CRT > 375 μm group was 4.06 months, whereas the median time to the second treatment in the initial CRT < 375 μm group was greater than 50 months (p = 0.022). **c** The median time to the second Ozurdex treatment in the diabetes group was 3.93 months, whereas the median time to the second treatment in the patients without diabetes was greater than 50 months (p = 0.003). More than 60% of the patients without diabetes only needed a single injection during the follow-up period, which was a statistically significant difference from the patients with diabetes (*p* = 0.003)

We performed a further Kaplan–Meier analysis stratified by age and found that the median time to the second Ozurdex treatment in the multiple-injection patients aged more than 60 years old was 3.96 months, whereas the median time in the patients aged less than 60 years old was greater than 50 months (*p* = 0.007) (shown in Fig. 2a). Furthermore, more than 70% of the patients younger than 60 years old only needed one injection during the follow-up period.

With respect to the initial CRT, we stratified the patients by initial CRT > 375 μm versus initial CRT < 375 μm. The median time to the second Ozurdex treatment in the initial CRT > 375 μm group was 4.06 months, whereas the median time in the initial CRT < 375 μm group was greater than 50 months (*p* = 0.022) (shown in Fig. 2b). In addition, more than 60% of the patients in initial CRT < 375 μm group only needed a single injection during the follow-up period.

Diabetes mellitus was also revealed to be an important risk factor. The median time to the second Ozurdex treatment in the patients with diabetes was 3.93 months, whereas the median time in the patients without diabetes was greater than 50 months (*p* = 0.003). Furthermore, more than 60% of the patients without diabetes only needed a single injection during the follow-up period, which was a statistically significant difference from the patients with diabetes (p = 0.003) (shown in Fig. 2c).

A multivariate Cox proportional hazard analysis was also performed, and its results also indicated that age, diabetes, and initial CRT were significant risk factors for additional treatment (Table 3). The other initial independent variables originally selected for the Cox proportional hazard model were gender, hypertension, phakic status, initial IOP, and initial BCVA, but these variables were excluded after model selection by stepwise method with the stay and entry significance level set at 0.05.

**Table 3:** Cox Proportional Hazards Analysis - Risk Factors for Additional Treatment

Parameter	Estimate	Standard Error	Chi-Square	Pr > ChiSq	Hazard Ratio	95% Confidence Limits
Age	0.0694	0.0294	5.5618	**0.0184**	**1.072**	1.012–1.136
Diabetes	1.7433	0.6241	7.8019	**0.0052**	**5.716**	1.682–19.425
Initial CRT	0.0068	0.0021	10.2207	**0.0014**	**1.007**	1.003–1.011

Multivariate logistic regression also verified that age was the most significant risk factor for additional treatment (for every one year, the risk of requiring an additional injection increased by 11.6%) (Table 4). The initial independent variables originally selected for the model in addition to age were diabetes, gender, hypertension, phakic status, initial IOP, initial BCVA, and initial CRT, but these variables were excluded after model selection by stepwise method with the stay and entry significance level set at 0.05 (AUC: 0.8029).

**Table 4:** Multivariate Logistic Regression - Dependent Variable: Single Injection vs. Multiple Injections (Event)

Parameter	DF	Estimate	Standard Error	Wald Chi-Square	Pr > ChiSq	Odds Ratio	95% Wald Confidence Limits
Intercept	1	−6.1763	2.6832	5.2983	0.0213		
Age	1	0.1095	0.0454	5.8174	**0.0159**	**1.116**	1.021–1.219

IOP elevation is an important concern in patients receiving an Ozurdex injection. The mean elevation of the peak IOP for all the patients in this study was from 15.3 ± 3.3 to 22.8 ± 7.2 mmHg (*p* < 0.0001). Among all 29 patients, 24.1% had a peak IOP > 25 mmHg and 10.3% had a peak IOP > 35 mmHg. The mean final IOP was 16.3 ± 3.0 mmHg, which showed no statistically significant difference from the mean initial IOP (*p* = 0.77). At the final visit, no patient had an IOP greater than 25 mmHg.

Cataract progression is another issue of concern. Significant lens opacity progression was found in 4 patients (19%) during the study period. Vision-impairing cataracts were not observed in the patients who only received one Ozurdex injection. However, in the multiple-injection group, the rate of lens opacity progression was relatively high at 40% after the second Ozurdex injection. In addition, 2 patients (both in the multiple-injection group) underwent cataract extraction surgery during the study period.

## Discussion

BRVO can lead to ME through an inflammatory process [14]. Specifically, BRVO has been found to reduce macular capillary blood flow, leading to the hypoxia-inducible factor-1a cascade [15, 16], the upregulation of endothilin-1 and VEGF, and, consequently, the breakdown of the blood-retinal barrier [1]. This breakdown causes a number of further consequences, such as increased vascular permeability and retinal hypoxia, that ultimately result in impaired vision.

A large number of studies have shown that intravitreal steroid injections are effective for treating BRVO-ME [17–23]. The effectiveness of such steroid injections stems from their anti-inflammatory properties and their capacity to stop the release of VEGF. Moreover, previous studies have proven Ozurdex to be a well-tolerated, efficacious treatment [17–23]. More specifically, the relatively long half-life of Ozurdex may yield substantial benefits compared to other treatments by allowing for a significantly longer interval between injections and, relatedly, better patient compliance [19].

Nonetheless, some patients do eventually require retreatment with Ozurdex due to recurrent ME. As such, this study was conducted to clarify the effects, duration of action, and safety of Ozurdex in treating BRVO-ME, as well as the risk factors for repeated treatment.

The results showed that Ozurdex provides significant functional improvements and concomitant anatomical responses in BRVO-ME patients. The efficacy and safety of Ozurdex in this study were comparable to those found in a recent randomized, sham-controlled, multi-centre study conducted in China [24], although the effects of Ozurdex lasted much longer in the present study.

In the COBALT prospective study, the retreatment schedule was set as 3 injections at 4-month intervals. The retreatment rate in that study was similar to that for our study (55.6% versus 51.7%). The mean changes in BCVA (+ 15.3 versus + 28 letters) and CRT (− 196 versus − 180 μm) from the baseline to 12 months after were also comparable [25]. Meanwhile, the relatively long treatment-free period in this study suggests that Ozurdex is more beneficial than other short-duration therapies.

With respect to treatment schedule protocols, most previous studies simply provided the treatment on an “as needed” basis. In the GENEVA study, the BCVA outcomes indicated that the peak efficacy occurred at day 60, whereas by day 180, the BCVA results of the Ozurdex-treated patients were no longer consistently significantly better than those of the sham group [17]. Furthermore, the mean change in CRT from the baseline in the GENEVA study was significantly better at day 90 but not day 180. In the aforementioned China Ozurdex in RVO study, Ozurdex showed similar efficacy outcomes with sham groups at months 5 and 6, suggesting the need for a re-treatment interval of 4–5 months for many patients [24]. Relatedly, recent studies have typically reported an average interval between injections of less than 6 months.

However, no consensus has been reached about the “as needed” criterion, and to achieve satisfactory visual and anatomic outcomes after the intravitreal administration of Ozurdex, we set up more aggressive criteria in this study. Specifically, any visual decrease attributable to BRVO-ME and not to other ocular conditions with an increase in CFT in OCT of > 50 mm from the lowest recorded level were chosen as the retreatment criteria in the current study.

Previous studies had reported that older age and greater initial CRT constitute risks for Ozurdex retreatment among some populations. For example, in a previous study conducted in Taiwan of both BRVO and central RVO patients, Lin et al. found that older age and greater initial CRT are risk factors for multiple injection in such patients [26]. Similarly, the COBALT study conducted in Korea also found that older age and higher initial CRT were indicators for reinjection of Ozurdex in BRVO-ME cases. Specifically, both the mean age and mean initial CRT of the patients who needed 3 injections over the 12-month study period were significantly greater than those of the patients who needed only one injection [25]. The results of the present study support those earlier findings. Specifically, our results indicated that age was the most significant risk factor for additional treatment (with the risk rising by 11.6% for each additional year), and that the median time to the second Ozurdex treatment in those with an initial CRT > 375 μm was significantly shorter than that in those with an initial CRT < 375 μm. Age was also negatively correlated with the mean reduction of CRT for the whole study group.

Altunel et al. speculated that the solubility and release of dexamethasone in the vitreous might change with aging [27]. The negative effect of aging on the efficacy of Ozurdex treatment in BRVO may result from the involutional changes of retinal pigment epithelium and impaired retinal cell function, among other aging-related changes. In any case, we calculated the hazard ratio for additional treatment as 1.072 and the median additional treatment-free survival time as 3.96 months in patients > 60 years old.

Higher initial CRT was also found to be a significant risk factor for retreatment, as the median time to the second Ozurdex treatment in the patients with an initial CRT > 375 μm was only 4.06 months, compared to greater than 50 months in the patients with an initial CRT < 375 μm. The pathophysiology of ME after RVO is due to hypoxia in the affected structures, and there might be more inflammatory activities inducing more severe ME in patients with higher initial CRT.

Through the multivariate analysis, we further found that the risk of re-treatment in those with diabetes was significantly higher than the risk in those without diabetes. Several biochemical mechanisms in diabetic patients, such as increased VEGF production and oxidative stress, may aggravate ME in BRVO [28–30]. Moreover, hyperglycemic conditions, which are frequently seen in patients with diabetes, can result in more severe hypoxia and damage to the retina, which may then deplete Ozurdex more rapidly [29, 30]. In fact, in the present study, the median additional treatment-free survival time in patients with diabetes was only 3.93 months (*p* = 0.003), and over 80% required re-treatment.

At the same time, our results also confirmed the benefits of Ozurdex treatment in treatment-naïve BRVO-ME patients. Specifically, the patients exhibited significant improvements in their final CRT and BCVA values, with 62.1% experiencing a final BCVA improvement of more than 15 letters.

Moreover, the safety profile of Ozurdex for BRVO-ME demonstrated in this study was favourable and consistent with those reported in previous studies [24–26]. Considering the peak level, in the present study, only 24.1% of the patients ever had an IOP > 25 mmHg, and increases in IOP were generally controlled with topical medications. Such elevations were transient, and all of the patients’ IOPs returned to an acceptable range before the end of the follow-up period. In the MEAD study of Ozurdex treatment in patients with diabetic ME, the mean IOP was shown to peak at 1.5–3 months after treatment and then to decrease to baseline levels by 6 months after treatment [31]. More importantly, Ozurdex showed no cumulative effect of sequential implants on IOP and no increase in the frequency of IOP elevations after repeat treatment [27]. As expected, in our study, no patient in the Ozurdex-treated group required incisional glaucoma surgery.

One strength of the present study was its use of a prospective design (although some data was retrospectively collected) in a real-world setting. Second, all of the included patients were treatment-naïve and treated according to the same treatment/follow-up protocol, while the data was collected under strict criteria, study design factors which gave the results greater validity than they otherwise would have had. Finally, we clearly identified the risk factors for repeated injections by applying a number of different models in a more comprehensive way.

The potential limitations of this study included its relatively small sample size, retrospective nature, and lack of control group. However, several studies previously demonstrated similar conclusions [24–26]. The spontaneous recovery of visual acuity in patients with BRVO-ME within one year has been found to range from 18 to 41% [25]. The response rate in our study, meanwhile, was far higher. Also, relevant variables were excluded in the present study in a stepwise manner. The number of predictors remained 2, with a desired statistical power level of 0.8, probability level of 0.05, and anticipated effect size of 0.37. We could thus lower the minimum required sample size to 29, which was the number of patients included in our case series. Nevertheless, while it is reasonable to think that these statistical methods are valid in these situations, further large-scale prospective studies should be conducted to fully evaluate treatment efficacy and establish the optimal retreatment schedule for Ozurdex in groups with a high risk of recurrence.

## Conclusions

In conclusion, this study confirms that Ozurdex results in significant CRT and BCVA improvements in treatment-naïve BRVO-ME patients. However, older age, high initial CRT, and diabetes are significant risk factors for recurrent ME and may thus result in the need for repeated injections. Furthermore, given that some BRVO-ME cases resolve spontaneously, the comparative efficacy and safety of Ozurdex could be clarified even further by additional studies including sham or active controls.

## Data Availability

The datasets used and/or analysed during the current study are available from the corresponding author on reasonable request.

## Abbreviations

BRVO-ME: Branch retinal vein occlusion-induced macular edema
BCVA: Best-corrected visual acuity
CRT: Central retinal thickness
VEGFL: Vascular endothelial growth factor
OCT: Optical coherence tomography
IOP: Intraocular pressure
ETDRS: Early Treatment Diabetic Retinopathy Study

## References

[1] Jaulim A, Ahmed B, Khanam T, Chatziralli I (2013). Branch retinal vein occlusion: epidemiology, pathogenesis, risk factors, clinical features, diagnosis, and complications. An update of the literature. Retina.

[2] Rogers S, McIntosh RL, Cheung N, Lim L, Wang JJ, Mitchel P (2010). The prevalence of retinal vein occlusion: pooled data from population studies from the United States, Europe, Asia, and Australia. Ophthalmology.

[3] Kolar P (2014). Risk factors for central and branch retinal vein occlusion: a meta-analysis of published clinical data. J Ophthalmol.

[4] Sivaprasad S, Amoaku WM, Hykin P, RVO Guideline Group (2015). The Royal College of ophthalmologists guidelines on retinal vein occlusions: executive summary. Eye (Lond).

[5] Morley MG, Heier JS, Yanoff M, Duker JS (2009). Venous obstructive disease of the retina. Ophthalmology.

[6] Department of Economic and Social Affairs Population Division (2004). World population to 2300.

[7] Laouri M, Chen E, Looman M, Gallagher M (2011). The burden of disease of retinal vein occlusion: review of the literature. Eye (Lond)..

[8] Rentz AM, Kowalski J, Revicki D, Loewenstein A, Blumenkranz MS, Yoon Y (2010). Normative comparison of generic-and vision-targeted health-related quality of life (HRQL) outcomes in patients with vision loss due to macular edema following retinal vein occlusion. Invest Ophthalmol Vis Sci.

[9] Chang MA, Fine HF, Bass E, Bressler SB, Schachat AP, Solomon SD (2007). Patients’ preferences in choosing therapy for retinal vein occlusions. Retina..

[10] Grzybowski A, Told R, Sacu S, Bandello F, Moisseiev E, Loewenstein A, Schmidt-Erfurth U, on behalf of the Euretina Board (2018). 2018 update on intravitreal injections: Euretina expert consensus recommendations. Ophthalmologica..

[11] Michelle W, Adrienne S (2013). How to give Intravitreal injections.

[12] Wang D, Amoozgar B, Porco T, Wang Z, Lin SC (2017). Ethnic differences in lens parameters measured by ocular biometry in a cataract surgery population. PLoS One.

[13] Gregori NZ, Feuer W, Rosenfeld PJ (2010). Novel method for analyzing snellen visual acuity measurements. Retina..

[14] Scholl S, Augustin A, Loewenstein A, Rizzo S, Kupperman B (2011). General pathophysiology of macular edema. Eur J Ophthalmol.

[15] Yamaguchi Y, Otani T, Kishi S (2006). Serous macular detachment in branch retinal vein occlusion. Retina..

[16] Fraenkl SA, Mozaffarieh M, Flammer J (2010). Retinal vein occlusions: the potential impact of a dysregulation of the retinal veins. EPMA Journal.

[17] Haller JA, Bandello F, Belfort R, Blumenkranz MS, Gillies M, Heier J, Loewenstein A, Yoon YH, Jacques ML, Jiao J, Li XY, Whitcup SM (2010). Randomized, sham-controlled trial of dexamethasone intravitreal implant in patients with macular edema due to retinal vein occlusion. Ophthalmology.

[18] Haller JA, Bandello F, Belfort R, Blumenkranz MS, Gillies M, Heier J (2011). Dexamethasone intravitreal implant in patients with macular edema related to branch or central retinal vein occlusion twelve-month study results. Ophthalmology.

[19] Moisseiev E, Goldstein M, Waisbourd M, Barak A, Loewenstein A (2013). Long-term evaluation of patients treated with dexamethasone intravitreal implant for macular edema due to retinal vein occlusion. Eye..

[20] Capone A, Singer MA, Dodwell DG, Dreyer RF, Oh KT, Roth DB (2014). Efficacy and safety of two or more dexamethasone intravitreal implant injections for treatment of macular edema related to retinal vein occlusion (Shasta study). Retina..

[21] Sheu SJ, Wu TT, Horng YH (2015). Efficacy and safety of dexamethasone intravitreal implant for treatment of refractory macular edema secondary to retinal vein occlusion in Taiwan. J Ocul Pharmacol Ther.

[22] Elbay A, Ozdemir H, Koytak A, Melikov A (2017). Intravitreal dexamethasone implant for treatment of serous macular detachment in central retinal vein occlusion. J Ocul Pharmacol Ther.

[23] Garweg JG, Zandi S (2016). Retinal vein occlusion and the use of a dexamethasone intravitreal implant (Ozurdex®) in its treatment. Graefes Arch Clin Exp Ophthalmol.

[24] Li X, Wang N, Liang X, Xu G, Li XY, Jiao J (2018). Safety and efficacy of dexamethasone intravitreal implant for treatment of macular edema secondary to retinal vein occlusion in Chinese patients: randomized, sham-controlled, multicenter study. Graefes Arch Clin Exp Ophthalmol.

[25] Yoon YH, Kim JW, Lee JY, Kim IT, Kang SW, Yu HG, Koh HJ, Kim SS, Chang DJ, Simonyi S (2018). Dexamethasone intravitreal implant for early treatment and retreatment of macular edema related to branch retinal vein occlusion: the multicenter COBALT study. Ophthalmologica..

[26] Lin CJ, Chen HS, Su CW, Tien PT, Lin JM, Chen WL, Kuo CY, Lai CT, Tsai YY (2017). The effect of age and initial central retinal thickness on earlier need of repeat Ozurdex treatment for macular edema due to retinal vein occlusion: a retrospective case series. J Ocul Pharmacol Ther.

[27] Altunel O, Göktaş A, Duru N, Ozkose A (2016). The effect of age on dexamethasone intravitreal implant (Ozurdex®) response in macular edema secondary to branch retinal vein occlusion. Semin Ophthalmol.

[28] Rahman S, Rahman T, Ismail AAS, A Rashid AR. (2007). Diabetes-associated macrovasculopathy: pathophysiology and pathogenesis. Diabetes Obes Metab.

[29] Collinson DJ, Rea R, Donnelly R (2004). Vascular risk: diabetes. Vasc Med.

[30] Huysman E, Mathieu C (2009). Diabetes and peripheral vascular disease. Acta Chir Belg.

[31] Maturi RK, Pollack A, Uy HS, Varano M, Gomes AMV, Li XY, Cui H, Lou J, Hashad Y, Whitcup SM, Ozurdex MEAD Study Group (2016). Intraocular pressure in patients with diabetic macular edema treated with dexamethasone intravitreal implant in the 3-year MEAD study. Retina..

